# A Novel Window into Angiogenesis—Intravital Microscopy in the AV-Loop-Model

**DOI:** 10.3390/cells12020261

**Published:** 2023-01-09

**Authors:** Ravikumar Vaghela, Andreas Arkudas, Daniel Gage, Carolin Körner, Stephan von Hörsten, Sahar Salehi, Raymund E. Horch, Maximilian Hessenauer

**Affiliations:** 1 Department of Plastic and Hand Surgery, University Hospital of Erlangen, Friedrich-Alexander Universität Erlangen-Nürnberg (FAU), 91054 Erlangen, Germany; 2 Department of Materials Science and Engineering for Metals, Friedrich-Alexander Universität Erlangen-Nürnberg (FAU), 91058 Erlangen, Germany; 3 Department of Experimental Therapy, University Hospital of Erlangen and Preclinical Experimental Animal Center, Friedrich-Alexander Universität Erlangen-Nürnberg (FAU), 91054 Erlangen, Germany; 4 Department of Biomaterials, University of Bayreuth, 95447 Bayreuth, Germany

**Keywords:** intravital microscopy, arteriovenous loop, tissue engineering, GelMA

## Abstract

Due to the limitations of current in vivo experimental designs, our comprehensive knowledge of vascular development and its implications for the development of large-scale engineered tissue constructs is very limited. Therefore, the purpose of this study was to develop unique in vivo imaging chambers that allow the live visualization of cellular processes in the arteriovenous (AV) loop model in rats. We have developed two different types of chambers. Chamber A is installed in the skin using the purse sting fixing method, while chamber B is installed subcutaneously under the skin. Both chambers are filled with modified gelatin hydrogel as a matrix. Intravital microscopy (IVM) was performed after the injection of fluorescein isothiocyanate (FITC)-labeled dextran and rhodamine 6G dye. The AV loop was functional for two weeks in chamber A and allowed visualization of the leukocyte trafficking. In chamber B, microvascular development in the AV loop could be examined for 21 days. Quantification of the microvascular outgrowth was performed using Fiji-ImageJ. Overall, by combining these two IVM chambers, we can comprehensively understand vascular development in the AV loop tissue engineering model¯.

## 1. Introduction

The reconstruction of large tissue losses that exceed the potential for natural regeneration remains one of the most challenging tasks in reconstructive surgery [1,2,3]. The success of free microvascular tissue transfer for reconstruction relies on factors such as the availability of healthy autologous tissue and donor site morbidities [2]. Tissue engineering enables the construction of bioartificial tissues from scratch by combining synthetic and biological materials. Therefore, it can efficiently address the problem of the human body’s limited supply of transplantable tissue. Consequently, the concept of tissue engineering has gained increasing acceptance among scientists and surgeons [1,2]. Essentially, tissue engineering allows the rational utilization of available transplantable tissue for the effective treatment of large tissue defects while maintaining functioning tissue.

However, a major challenge for any tissue-engineered construct is the generation of adequate vasculature (in the entire 3D construct) and the immediate supply of the required nutrients and oxygen [4,5]. Moreover, the lack of of immediate perfusion to the generated bioartificial transplants could cause complications such as wound healing disorders and tissue necrosis [6,7]. Therefore, any successful tissue engineering-based fabrication of tissue constructs requires a thorough understanding of vascular biology. Only a detailed understanding of vascular biology for tissue engineering applications can guarantee the success of a large-scale tissue engineering approach.

Furthermore, there is a lack of in vivo study models suitable for a detailed understanding of vascular development. In vitro and ex vivo models have demonstrated limited pre-clinical compatibility [8,9]. Moreover, due to the limitations of the current in vivo experimental design, our comprehensive knowledge of vascular development and its implications for the development of large-scale engineered tissue constructs is very limited. The conventional experimental approach limits our understanding to one static point, which can only be generated after several processing steps (histology staining, polymerase chain reaction (PCR), etc.) [10,11,12].

This limited knowledge is mainly due to the lack of an experimental setting focused on the IVM (intravital microscopy) approach. In contrast to the conventional experimental approach, IVM allows the repeated study of cellular events in the native environment of a living animal [13]. The IVM technique offers a comprehensive perspective of the entire cellular event with less animal exploitation and less intra-animal variation [10].

Therefore, this study focused on the development of unique in vivo imaging chambers for imaging the ongoing cellular events in the AV loop model of rats. The AV loop model (first published by Erol and Spira) has been proven to be the most viable tissue engineering approach for the in vivo generation of axially vascularized large tissue constructs [11,14,15,16,17].

This manuscript summarizes our efforts to establish IVM in the AV loop and evaluate its value for assessing vascular development in the AV loop. We have developed several different chamber designs [18,19]. Chamber design A allows repeated short-term (direct) imaging of the initial dynamic event following AV loop surgery, while chamber design B enables periodic longitudinal assessment of the vascularization and de novo tissue generation.

## 2. Materials and Methods

### 2.1. Animals

We performed AV loop experiments on ten male Lewis rats acquired from Charles River (Sulzfeld, Germany). All animal experiments were carried out in accordance with German animal welfare laws. Moreover, all the surgical intervention protocols applied in this study were approved by the Regierung von Unterfranken, Würzburg, Germany, under license number 55.2-2532-2-519. All animals used in this study were kept at the Franz Penzoldt Zentrum (FPZ) of the Friedrich Alexander Universität (FAU), Erlangen-Nürnberg.

Prior to the surgery, all animals were quarantined for at least a week, and implantations were performed in animals weighing between 400 and 460 g (gm). After AV loop surgery, animals were maintained in individual cages in a standardized environment with free access to tap water and laboratory food (ssniff, Spezialdiaeten GmbH, Soest, Germany) until the end of the experiment.

### 2.2. Chamber Design

We developed two different chamber designs: design A and design B. The chambers were made of titanium (Ti) alloy powder titanium–6aluminum–4vanadium (Ti-6Al-4V, TEKNA Advanced Materials Inc., Sherbrooke, QC, Canada). The 3D fabrication process of the chambers was carried out at the Department of Materials Science and Engineering for Metals, FAU, Erlangen using selective electron beam melting (SEBM). The resulting 3D objects were further processed using a Computer Numerical Control (CNC) mill.

**Figure 1:**
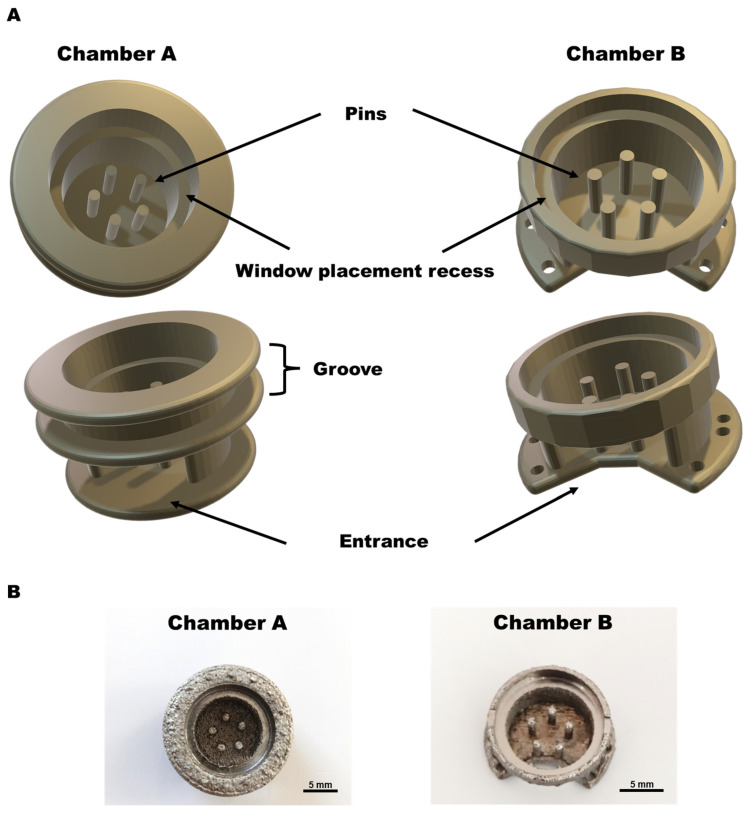
Chamber designs. (**A**) A CAD illustration of chamber designs. (**B**) Chambers after CNC processing. Chambers are produced as single-piece designs containing a spherical base chamber with five pins in the middle for retaining the AV loop and a circular recess on the top part of the chamber to accommodate the circular glass window. Chamber A contains an additional groove for fixing the chamber to the skin. Scale bar: 5 mm. Additional 3D design sketches in Supplementary Material.

Both chamber types have the same fundamental structure, which consists of a ring-shaped chamber with five pins to retain the AV loop inside the chamber. Additionally, it has a recess for the placement of the spherical (12 mm ø) observation window (Hecht-Assistent, Sondheim vor der Rhön, Germany). A snap ring (Würth, Bad Mergentheim, Germany) is used to secure the viewing window (Figure 1, Supplementary Material).

However, both chambers have slightly different initial surgical preparations and are fixed with different techniques. Chamber A contains a groove, and it is fixed to the skin using the purse string method, allowing direct access to the chamber for imaging without surgical exposure, whereas chamber B is fixed securely under the skin.

### 2.3. Surgical Procedure

The AV loop surgery was performed as previously described [1,2]. Briefly, the animal is first anesthetized (with oxygen and isoflurane, Baxter, Germany) and then placed on a 37 °C heating plate under the surgical microscope (Karl Zeiss, Jena, Germany). For pain management, 1.5 mg/kg of butorphanol and 0.5 mg/kg of meloxicam are injected intravenously, and antibiotics (3 mg/kg arbofloxacin) are given intravenously via the tail vein. In addition, eye ointment Bepanthen^®^ (Bayer AG, Leverkusen, Germany) is applied to keep eyes from drying.

Then, both hind limbs are disinfected with alcohol, and a mid-ventral skin incision is made on the left leg to expose the femoral vascular bundle. Both chambers are fixed with different techniques at the end of surgery. Therefore, different types of skin incisions are performed for both chambers. For chamber design A, a horizontal incision is performed and for chamber design B, a vertical incision is performed.

Then, a longitudinal incision is made on the contralateral side, and the femoral vein is carefully exposed. The interpositional venous graft (IVG) is collected from the donor leg. Following that, the recipient femoral vein is temporarily ligated using microvessel clamps at the proximal end and the distal end is cut with micro scissors. Both the IVG graft and recipient vein are then flushed with an anticoagulant solution (50 IU/mL heparin).

The proximal end of the IVG is then sutured (8–10 interrupted sutures) to the distal end of the left femoral vein with microsutures (Ethilon 11–0; Johnson & Johnson, New Brunswick, NJ, USA). Similarly, the recipient femoral artery is separated at the distal end and connected with the distal end of IVG to create a complete AV loop. A 3 mm-long intravascular stent made of prolene suture material (Prolene 4–0, Johnson & Johnson Medical GmbH, Norderstedt, Germany) is used to ensure smooth anastomosis between the femoral vessels [20,21].

Once the AV loop is created, it is carefully checked for patency and any leaks. A steam-sterilized titanium chamber is then pre-filled to three-quarters of its volume with a 10 wt.% (100 mg/mL) methacrylate gelatin (GelMA) matrix. The 10 wt.% GelMA solution is prepared by dissolving 100 mg of GelMA lyophilized hydrogel at 37 °C in PBS. Then, 10 mg of crosslinker Lithiumphenyl-2,4,6-trimethyl-benzoyl phosphinate (LAP, Sigma-Aldrich, Burlington, MA, USA) is added as a photoinitiator. The pre-filled GelMA matrix in the chamber is crosslinked using exposure to UV light (395–400 nm; 80–150 mcd) for 30 s (UV-lamp, EFL41UV UV, Perel, Gavere, Belgium).

The chamber is carefully placed and fixed to the underlying muscle using a 6–0 prolene suture. The AV loop is then carefully placed in the chamber on the crosslinked GelMA without causing any unwanted stretching or kinking. It is important that the loop is as close to the coverslip as possible to ensure quality in intravital imaging. Subsequently, the GelMA matrix is cast on top of the AV loop and the loop pedicle, as well as around the chamber, followed by further crosslinking using the UV lamp.

**Figure 2:**
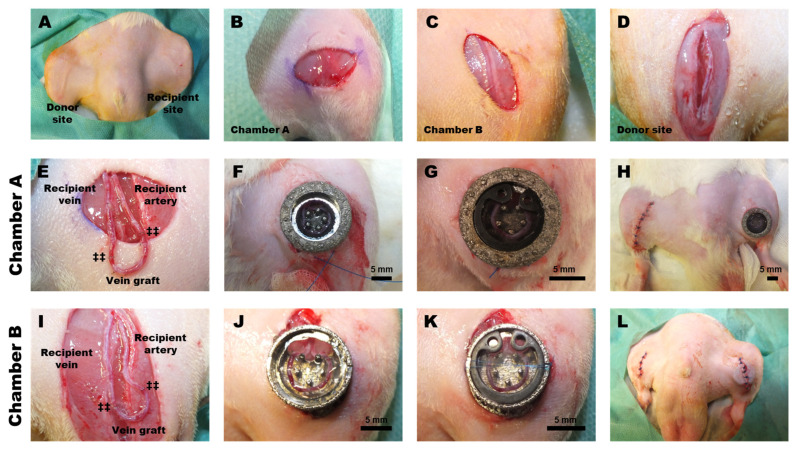
AV loop preparation. (**A**) Shaving and disinfection of the donor as well as recipient sites. (**B**) For chamber A surgical preparation, a small horizontal incision is applied to open the skin. (**C**) For chamber B the skin is opened by a vertical incision. (**D**) Next, the donor femoral vein is exposed and carefully separated. (**E**) AV loop preparation for chamber A. (**F**) After positioning a loop in chamber A (partially filled with GelMA), the skin is retained in the grove, and the chamber is fastened to the skin using a purse string fixing technique. (**G**) Placement of the glass window with a snap ring and chamber installation. (**H**) Top view of the surgical site following the AV loop procedure. (**I**) AV loop preparation for chamber B. (**J**) Placement of the loop in the partially GelMA-filled chamber B. (**K**) Placement of the glass window. (**L**) Top view of the surgical site after subcutaneous installation of chamber B. ‡‡: microsurgical anastomosis; scale bar: 5 mm.

Then the glass window is fixed to the chamber with a snap ring. It is important to eliminate any unwanted air bubbles. In the case of chamber A, the skin is first carefully placed in the chamber groove and then fixed using a purse string fixing technique (with 4–0 suture). The top part of chamber design A is left above skin level to allow direct imaging from the chamber. On the other hand, design B is fixed subcutaneously (s.c.) under the skin. The surrounding skin is closed on the top of design B with two layers of suturing (Figure 2).

### 2.4. Post-Operative Treatment

To ensure animal well-being, each operated animal was screened for their health three times daily for the first three days and then once a day until the end of the study. The animals received follow-up pain management medications, an anti-coagulant, and an antibiotic for the first week after the surgery.

For the first three days, each animal received butorphanol (1.5 mg/kg) and meloxicam (0.5 mg/kg), as well as buprenorphine (0.05 mg/kg at night) for pain management, antibiotic marbofloxacin (3 mg/kg), and anti-coagulant enoxaparin (10 mg/kg). The animals received daily meloxicam (0.5 mg/kg) for the rest of the week.

### 2.5. Intravital Microscopy

The glass window in chamber A remained above skin level and was therefore freely accessible for frequent imaging without the need for surgical intervention. On the other hand, chamber B was installed under the skin. Therefore, minimally invasive surgical exposure of the chamber was necessary.

Before imaging, the animal was once again anesthetized using isoflurane with oxygen. For staining the blood plasma, the animal was injected with a 5% solution of FITC-labeled dextran (0.5 mL, Sigma-Aldrich, Burlington, MA, USA; MW 500,000) into the tail vein. To examine leukocyte recruitment, a 0.1 wt.% solution of Rhodamine 6 G (0.6–1.0 mL, Sigma-Aldrich, Burlington, MA, USA) dye was injected.

The glass window was carefully cleaned with Q-tips (Lohmann & Rauscher, Rengsdorf, Germany), and then the animal was positioned under the microscope with continuous anesthesia. For intravital imaging, a modified upright Nikon 80i epifluorescence microscope (Nikon, Tokyo, Japan) with an X-Cite 120 mercury light source (Olympus, Tokyo, Japan) was used. Intravital images were gained from each region of interest (ROI) containing microvascular sprouting.

In the case of chamber B, surgical closure of the skin after imaging was required. After completion of the imaging process, the animal was kept on a heated surface until it regained consciousness.

### 2.6. Microcirculatory Analysis

**Figure 3:**
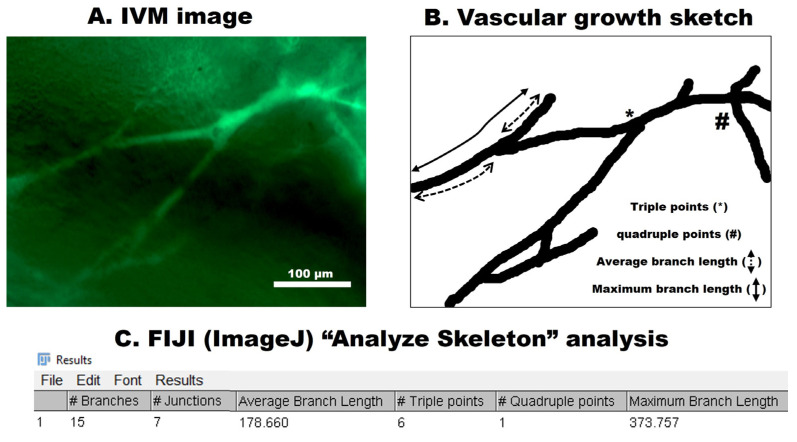
Analysis of the IVM images. (**A**) A representative IVM image (HPF) showing fluorescent microvessels. (**B**) An overlay micrograph (vascular growth sketch) of the IVM image prepared using GIMP software. (**C**) Representative results of vascular growth sketch analysis in Fiji-ImageJ using the “Analyze Skeleton” plugin. Scale bar 100 μm.

Neovessel outgrowth from the AV loop was analyzed as described previously [18,19]. Briefly, overlaid vessel network sketches for each High-Power Field (HPF) were prepared using the GNU Image Manipulation Program (GIMP). Later, each sketch was processed using Fiji-ImageJ (NIH, Bethesda, MD, USA) and then analyzed using the “Analyze Skeleton” plugin. The obtained results were further analyzed for the quantification of the vascular outgrowth (Figure 3).

### 2.7. Statistical Analysis

The normal distribution of the IVM data was examined with the Shapiro–Wilk test as well as the Kolmogorov–Smirnov test. Two-group comparisons were performed with an unpaired Student’s *t*-test. Comparisons between more than two groups were evaluated with one-way ANOVA. Data are expressed as median with an interquartile range. Statistical significance was defined as *p* values < 0.05.

## 3. Results

### 3.1. Chamber Stability

In our previous study, we identified several problems relating to the stability of the skin around the chamber and manipulation [18]. Therefore, each animal in this study was examined daily in detail for its health and chamber stability.

Chamber A contained a U-shaped groove in the upper part of the chamber. The chamber was implanted into the skin of the animal through a purse string suture. The purse string suture kept chamber A stable on the animal’s leg for a period of up to 10–14 days. We were able to establish chamber stability for up to 30 days by repeating the purse string suture when required.

Chamber B was designed for subcutaneous fixation. Numerous previous (non-IVM) studies have shown that a subcutaneously fixed chamber with a functional AV loop can remain stable for at least 8 weeks [22]. In this study, chamber B needed to be exposed surgically for each imaging session and was once again secured beneath the skin. This surgical exposure of the chamber did not affect the stability of the chamber. Chamber B remained stable until the end of the study period.

### 3.2. Intravital Imaging

Fluorescence dye was injected for performing intravital imaging of the AV loop. FITC-Dextran was used to stain the blood plasma and Rhodamine 6G was used to examine rolling circulatory cells.

Chamber A allowed frequent imaging of the AV loop chamber without the need for any surgery. Chamber A achieved AV loop patency for up to 14 days. Chamber A allowed a thorough examination of the early tissue integration phase in the AV loop model. Using Rhodamine 6 G dye, we were able to visualize rolling leukocytes in the circulation as well as firmly attached leukocytes near anastomosis sites.

**Figure 4:**
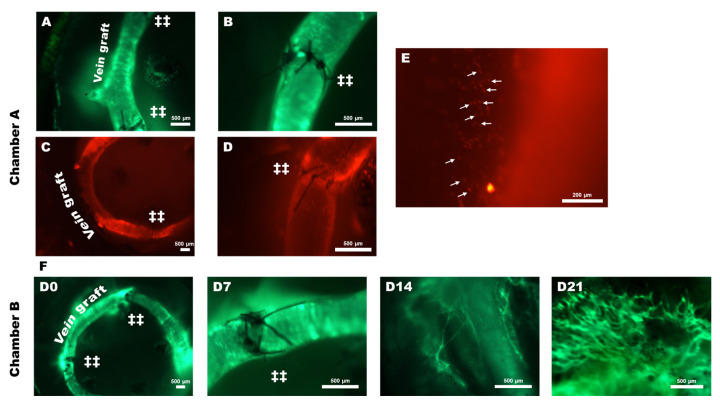
Chamber A IVM. AV loop vessels were visualized by injecting FITC-Dextran dye, which stains the blood plasma (**A**,**B**); scale bar: 500 μm. Leukocyte–endothelial cell interactions can be examined using rhodamine 6G (a pan-leukocyte marker) dye (**C**,**D**); scale bar: 500 μm. (**E**) A representative image showing firmly attached leukocytes to the vessel wall. Scale bar: 200 μm. (→ firmly adherent leukocytes). (**F**) Chamber B IVM was performed on days 0, 7, 14, and 21. No microvessels were observed on day 7. On day 14, vascular sprouts were first observed. Microvessels were also observed on day 21. Scale bar 500 μm. (‡‡: microsurgical anastomosis).

In the case of chamber B, IVM was performed on days 0 (before subcutaneous chamber fixing), 7, 14, and 21. Microvessels sprouting from the AV loop were visualized by injecting the contrasting agent FITC-Dextran through the tail vein. Fluorescent microvascular structures that emerged from the AV loop were characterized as microvessels. Visible neovascular sprouting was observed on day 14 and day 21. No apparent microvascular outgrowth was observed during the first week after the operation (Figure 4).

### 3.3. Intravital Analysis

**Figure 5:**
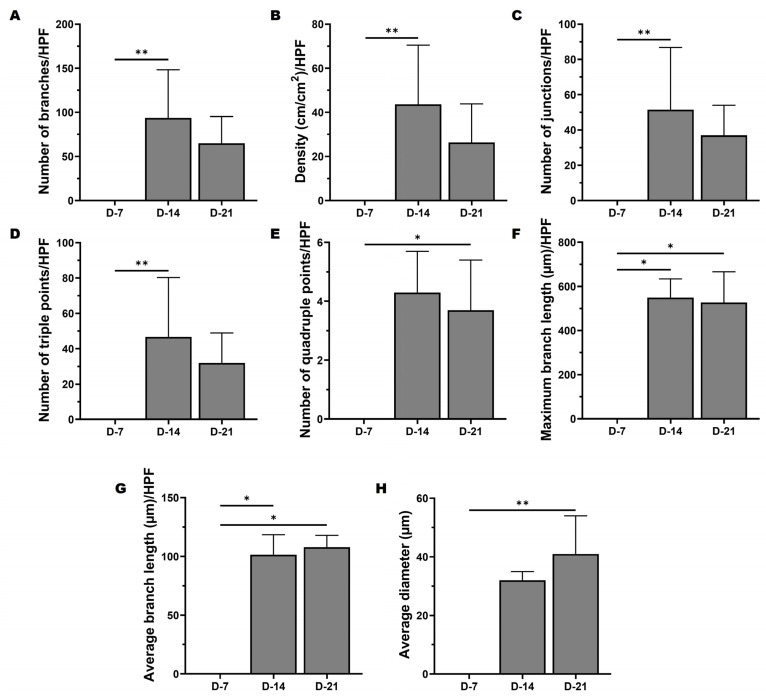
Chamber B IVM analysis. (**A**) Number of branches. (**B**) Density (cm/cm^2^). (**C**) Number of junctions. (**D**) Number of triple points. (**E**) Number of quadruple points. (**F**) Maximum branch length. (**G**) Average branch length (μm) per HPF and (**H**) average vascular diameter (μm) were calculated. Data are presented as medians with interquartile ranges. Significance at * *p* < 0.05 and ** *p* < 0.01.

Intravital images obtained from chamber B were further processed with GIMP software, followed by Fiji-ImageJ analysis. Functional microvessels sprouting from the AV loop were observed on days 14 and 21, as reported previously [19]. Therefore, these images were further analyzed for the quantification of vascular outgrowth (Figure 5).

On day 14, there was a statistically significant increase in vascular outgrowth (number of branches, 0.006, and vascular density cm/cm^2^, *p* = 0.006) compared to day 7. In addition, vascular interconnectivity was determined based on the number of junctions, triple points, as well as quadruple points. A significant increase in the number of junctions (*p* = 0.005) and the number of triple points (*p* = 0.004) was observed on day 14. On the other hand, a significant number of quadruple points (*p* = 0.029) was observed on day 21 in comparison to day 7. Interestingly, a slight drop in vascular growth was detected on day 21.

Moreover, a statistically significant expansion of average branch length was detected on day 14 (*p* = 0.019) and day 21 (*p* = 0.023) over day 7. Likewise, a significantly higher maximum branch length was noted on day 14 (*p* = 0.012) and day 21 (*p* = 0.035) per HPF compared to day 7. Notably, on day 21, there was a significant statistical rise in the overall diameter of microvessels (*p* = 0.003) compared to day 7.

## 4. Discussion

Vascular tissue engineering promises to significantly influence a wide range of clinical conditions. Blood vessels are responsible for the supply of oxygen and nutrients as well as the removal of waste from the tissues [4,5]. Physical influences such as pressure, torque, or periods of ischemia are other factors known from clinical practice to interact with vasculogenesis and perfusion [7,23]. However, providing microvasculature to thick tissues (>100–200 microns) is one of the main obstacles in the field of tissue engineering [4]. Although in vitro and ex vivo studies provide valuable results, tissue engineering mainly relies on performing in vivo experiments because of its clinical relevance [8,9,24].

Moreover, currently available models provide a limited amount of information regarding in vivo vascular development and what role it plays in the overall tissue generation in the defective area. This restricted information is due to the limited implications of the IVM approach in tissue engineering experiments.

IVM is a unique in vivo imaging method that allows microscopic-level visualization inside living subjects. IVM imaging offers several advantages over conventional experimental setups. The IVM technique allows the observation of dynamic events at cellular and subcellular resolution using small animal models after performing minimally invasive procedures [10]. While conventional in vitro and ex vivo systems very loosely mimic in vivo conditions, IVM imaging allows the examination of cellular activity in the native in vivo condition. Moreover, surrounding vasculature, lymphatics, and nerve arrangements are maintained without any manipulation during IVM experiments [13,25].

The IVM model is compatible with both short-term and long-term studies. Moreover, repetitive imaging of the ROI is possible, allowing a complete overview of the undergoing dynamic cellular process without sacrificing an animal [10,13,26]. In conventional experiments, this can only be achieved by sacrificing animals at many end points and extracting organs of interest [10]. Then, various time-consuming processing steps must be performed in order to understand the final cellular or molecular outcome or development. The same can be achieved in the IVM setting by injecting specific antibodies or test molecules into a living animal. Importantly, the IVM setting also allows the extraction of the specimen of interest for performing detailed histological, molecular, or protein profiling analyses at the end of the study [5,19]. In this way, IVM results can be further supported by ex vivo examinations.

Moreover, multiple labeling methods have been developed that allow the tagging of one or multiple cell types simultaneously at a specific ROI [13]. Therefore, IVM allows us to study complete cellular interactions in an unmanipulated environment. Since a complete overview can be generated with a smaller number of animals, the IVM approach effectively contributes to obtaining high-quality information in a short time and in a more humane way [10,13,18,19].

One of the major advantages of IVM is that it provides in vivo imaging of various dynamic processes in 3D in living animals [13]. There are various optical methods available that enable layer-by-layer imaging at various penetration depths, which is not possible to achieve with the conventional observation of the 2D static histology samples [13,26]. Horton et al. [27] used three-photon microscopy (3 PM) for the deep imaging (penetration depth of 1.3 mm) of vascular and neuronal structures in the mouse brain with the help of a long excitation wavelength (1700 nm). Similarly, Wang et al. [28] used confocal fluorescence for the observation of the high endothelial venules (~6.6 μm Ø), and immune trafficking in inguinal mice lymph nodes (at a penetration depth of ~1.1 mm).

IVM also allows in vivo visualizations of cellular trafficking and interaction (cell-cell/microenvironment), gene expression, and protein activity [13,26,29]. Moreover, in vivo physiological responses to stimuli or molecules, as well as the biocompatibility of various matrices, can be determined.

As shown in our previous study, we can still extract in vivo specimens at the end of the IVM study. A detailed in vitro investigation (histology, molecular, or protein profiling) of the extracted specimen adds more evidence in support of the IVM findings [19].

IVM studies are very important in tissue engineering experiments to rapidly verify the suitability of implanted biomaterials. Hessenauer et al. [30] demonstrated that the surface coating of porous polyethylene (PPE) implants with matricellular proteins such as Vitronectin (VN) effectively accelerated and enhanced vascularization in the implant. This could be particularly beneficial for use in areas that are not ideal for implantation without compromising the integrity of the host tissue. In a similar study, Gniesmer et al. [31] showed that chitosan-graft PCL (CS-g-PCL) enhances the development of vascularized tissue and cell ingrowth into electrospun polycaprolactone (PCL) scaffolds. Recently, Weinzierl et al. [5] assessed the angiogenic potential of nanofat grafts in a murine IVM model. Intravital analysis, followed by histology and immunohistochemistry, demonstrated rapid vascularization and the formation of a functionally dense microvasculature in both male and female mice.

Fluorescence has evolved into the most commonly used contrast strategy in IVMs, thanks to a wide variety of molecular probes [13,26]. However, over time a variety of alternative endogenous contrast techniques have been developed that provide information without depending on fluorescence tagging, such as second harmonic generation (SHG), third harmonic generation (THG), and coherent anti-Stokes raman scattering (CARS) [10,32,33].

The AV loop is the most effective technique for the generation of in vivo, axially vascularized, and surgically compatible tissue [14,34,35,36,37]. Therefore, the rat AV loop model is the best model to study vascularization as well as de novo tissue generation. Depending on the chamber, this model enables both intrinsic and extrinsic vascularization. Moreover, a rational combination of specific cell types, growth or differentiation factors with a suitable matrix can be seeded in the chamber for specific tissue development [38,39,40,41]. The AV loop supports the generation of both vascularized bone tissue as well as soft tissue constructs [14,42,43,44].

In the conventional AV loop, the experimental design does not provide a comprehensive overview of the undergoing regeneration process. However, with our newly developed chambers A and B, a complete overview, starting from immune cell recruitment followed by endothelial cell engraftment, and conversion into specific tissue constructs, can be studied.

**Chamber A vs. chamber B**

In the following, we summarize and compare both chamber designs A and B. In chamber A, the glass window is open and freely accessible. Therefore, frequent imaging can be performed without the need for surgical exposure. Moreover, chamber A is fixed using the purse string method, which has several advantages. In this method only one knot is tied at the bottom of the chamber. In our previous experience, a higher number of knots resulted in several accessible threads that easily attracted the animal’s attention for manipulation. Furthermore, a single knot tied at the bottom of the chamber is not easily accessible for animals. Therefore, purse string fixing effectively eliminates the possibility of manipulation by the animal.

The implanted chambers should be examined regularly and thoroughly. In chamber A, the observation window (0.1–0.2 mm-thick coverslip) is located outside of the animals’ skin. Therefore, it gets dirty and can easily break when accidentally colliding with other objects. Before imaging, it needs to be properly and gently cleaned without putting excessive pressure on the loop and the loop pedicle. The lack of additional covering puts extra stress on the loop and the pedicle during any movement, which limits the loop’s functionality to 2 weeks. In chamber B, we successfully overcame this instability issue by first firmly tying down the chamber with a suture tread passing above the chamber and then securing the chamber underneath the skin.

In this study, we used the crosslinked GelMA hydrogel as a matrix. GelMA is a highly transparent, slow-degrading matrix, developed by adding methacrylate groups to gelatin’s amine-containing side groups [12,45,46]. Additionally, it allows in vivo vascularization and is a good choice for longitudinal studies [12]. In a previous study, we have shown that the in vivo matrix’s transparency slowly decreases, which affects imaging quality. Moreover, any faster-degrading matrix puts extra stress on the AV loop and the loop pedicle, which ultimately compromises the loop functionality [19]. Therefore, GelMA is a more appropriate choice for IVM experiments. Consequently, the degradation rate and the in vivo matrix transparency are important for the selection of the matrix.

## 5. Conclusions

In this study, we developed two different chamber designs A and B. Chamber design A allows direct imaging of the initial dynamic events following AV loop surgery. Chamber B allows periodic imaging of the vascular outgrowth after performing surgical exposure of the chamber. In combination, we can acquire a complete overview of the vascular development in the AV loop model, which can ultimately provide valuable information for the advancement in the field of tissue engineering as well as reconstructive surgery.

## Data Availability

Primary data are available from the corresponding author upon reasonable request.

## Supplementary Materials

The following supporting information can be downloaded at: https://www.mdpi.com/article/10.3390/cells12020261/s1, Video S1: Chamber A, Video S2: Chamber B.
